# Effects of the Combination of Auricular Acupressure and a Fluid-Restriction Adherence Program on Salivary Flow Rate, Xerostomia, Fluid Control, Interdialytic Weight Gain, and Diet-Related Quality of Life in Patients Undergoing Hemodialysis

**DOI:** 10.3390/ijerph181910520

**Published:** 2021-10-07

**Authors:** AeKyung Chang, YoonChung Chung, MoonJa Kang

**Affiliations:** 1 College of Nursing Science, Kyung Hee University, Seoul 02447, Korea; 2 Department of Nursing, Graduate School, Kyung Hee University, Seoul 02447, Korea; kinokira@naver.com (Y.C.); sinbong2001@hanmail.net (M.K.)

**Keywords:** acupressure, hemodialysis, xerostomia, fluid control, quality of life

## Abstract

Adherence to fluid-restriction is a clinical priority in nephrology care. This study examines the effects of a combination of auricular acupressure (AA) and a fluid-restriction adherence program on the salivary flow rate, xerostomia, fluid control, interdialytic weight gain (IDWG), and diet-related quality of life (DQOL) among hemodialysis patients in South Korea. Using a quasi-experimental design, 84 hemodialysis patients were assigned to the experimental group (AA + fluid-restriction adherence program; *n* = 29), the comparison group (fluid-restriction adherence program; *n* = 27), and the control group (usual care; *n* = 28). The program lasted 6 weeks, and data were collected at baseline, immediately after the intervention, and 4 months post-intervention. There was a significant interaction between group and time for salivary flow rate, fluid control, IDWG, and DQOL (all *p* < 0.005). Compared with the control group, the experimental group had a significantly improved salivary flow rate, fluid control, IDWG, and DQOL at weeks 6 and 22, whereas the comparison group had improved fluid control and DQOL at week 6. The combination of AA and a fluid-restriction adherence program could be provided to hemodialysis patients as cost-effective, safe, and complementary interventions to promote sustainable patient adherence to fluid-restriction.

## 1. Introduction

The adherence to fluid-restriction is a clinical priority in nephrology care [1]. Among the hemodialysis (HD) treatment regimens, fluid-restriction is reportedly the most challenging [2] and has the highest non-adherence rate [3,4]. Poor adherence to fluid-restriction can lead to interdialytic weight gain (IDWG), and increased IDWG has been associated with cardiovascular diseases, which is one of the major causes of mortality among patients on HD [5]. Based on consistent reports that a high IDWG results in supplementary dialysis sessions with increased costs and deterioration of the quality of life, and that a 1% increase in body weight is strongly associated with the increased risk of mortality in HD patients [1,6], effective and safe interventions are urgently needed to improve fluid-restriction adherence among this population.

Limiting fluid consumption is a complex part of treating end-stage renal disease (ESRD) as there are various factors that influence patients’ acceptance of this requirement [5]. Factors related to fluid-restriction adherence of HD patients include personal factors, such as thirst, xerostomia, and knowledge; psychological factors, such as confidence, self-control, and social support; and environmental factors, such as cultural patterns and education programs [4]. Furthermore, the National Kidney Foundation recommends a combined use of fluid control methods for facilitating adherence to fluid-restriction: alleviating xerostomia, tracking fluid intake, limiting sodium intake, and providing personalized fluid goals [7]. Taken together, a comprehensive fluid-adherence intervention that incorporates mitigation of xerostomia symptoms and increases self-monitoring, self-control, knowledge, and social support for successful fluid management is warranted for HD patients.

Empowerment models identifying factors related to decision-making for behavior change and have been used to facilitate self-management [8]. The components of such models include knowledge and skills acquisition, improvement of self-efficacy and self-control, a network of social support, and active participation, which can be utilized as intervention strategies to achieve desirable health goals. Further, while empowerment strategies have been used to improve self-efficacy and physiological parameters, such as IDWG, serum hemoglobin and hematocrit levels of HD patients [9], there is a lack of sufficient literature to support the effectiveness of empowerment strategies on fluid-restriction adherence in HD patients.

Xerostomia caused by reduced salivary production is a major obstacle to the adherence to fluid-restriction of HD patients [10,11]. While the unstimulated salivary flow rate in healthy people ranges between 0.3 and 0.5 mL/min, about half of HD patients have a rate below 0.1 mL/min [2], and 60–77% of the patients experience severe thirst and xerostomia [6]. Stimulation of the salivary glands by mechanical means such as transcutaneous electrical nerve stimulation (TENS) and acupuncture has been proven effective in alleviating xerostomia or promoting saliva secretion in HD patients [2,6]. As one form of salivary gland stimulation, auricular acupressure (AA) is based on the principle that specific points on the ear correspond to internal organs and body meridians and that stimulating these auricular acupoints can treat ailments of the internal organs [12]. Unlike TENS and acupuncture, AA is non-invasive, safe and can be self-performed following professional instruction. It has been used to manage discomfort in HD patients, such as sleep disorders, itching, and pain [13]. Further, compression of auricular acupoints related to saliva secretion activates the parasympathetic nervous system and increases the saliva flow rate, thereby reducing xerostomia [14]. Therefore, a fluid-restriction adherence intervention incorporating AA may be an alternative and viable method for HD patient care.

However, most of the existing studies for fluid management programs in HD patients have not reflected the complex nature of fluid-restriction, as they provided only educational or cognitive interventions, fluid intake monitoring, skill training, and diet counseling [15,16,17,18,19]. Based on the promising evidence for AA and the multifaceted characteristics of fluid-restriction adherence, this study was conducted to compare the saliva flow rate, xerostomia, fluid control, IDWG, and diet-related quality of life (DQOL) of HD patients receiving a either combination of AA and a fluid-restriction adherence program or a fluid-restriction adherence program to those of patients receiving the usual renal care.

## 2. Materials and Methods

### 2.1. Conceptual Framework

The intervention program was developed based on the empowerment model [8]. Empowerment refers to the process of an individual’s inner growth, which involves acquiring knowledge and skills, social support, self-control, self-efficacy, and active participation. These components led to the development of this combination of AA and a fluid-restriction adherence program, which included the following: (1) improvement of self-control and self-efficacy through individual counseling; (2) knowledge enhancement through group education; (3) social support and active participation through group discussion; and (4) skill acquisition via AA.

### 2.2. Design

A quasi-experimental pretest–posttest design was used. Measurements were taken at baseline, immediately after the 6-week intervention, and 4 months post-intervention.

### 2.3. Participants and Setting

Participants of the study were hemodialysis patients, recruited from three HD centers (A, B, and C) of three university hospitals based in Seoul, South Korea. We used a convenience sampling method for the HD centers and participants. The centers were similar in size, operation status, staff composition, and number of patients. To prevent between-participant interaction, the patients in center A were allocated to the experimental group (combined AA and fluid-restriction adherence program), those in center B to the control group (usual care), and those in center C to the comparison group (fluid-restriction adherence program). Inclusion criteria were as follows: patients aged ≥20 years, requiring HD three times a week for ≥6 months, and available for saliva measurement. Exclusion criteria were as follows: having a history of acute cardiovascular disease, Sjögren’s syndrome, ear infection, allergy to seeds of semen vaccaria or use of drugs that may induce xerostomia (e.g., anticholinergics, tricyclic antidepressants, and beta-blockers). 

**Figure 1:**
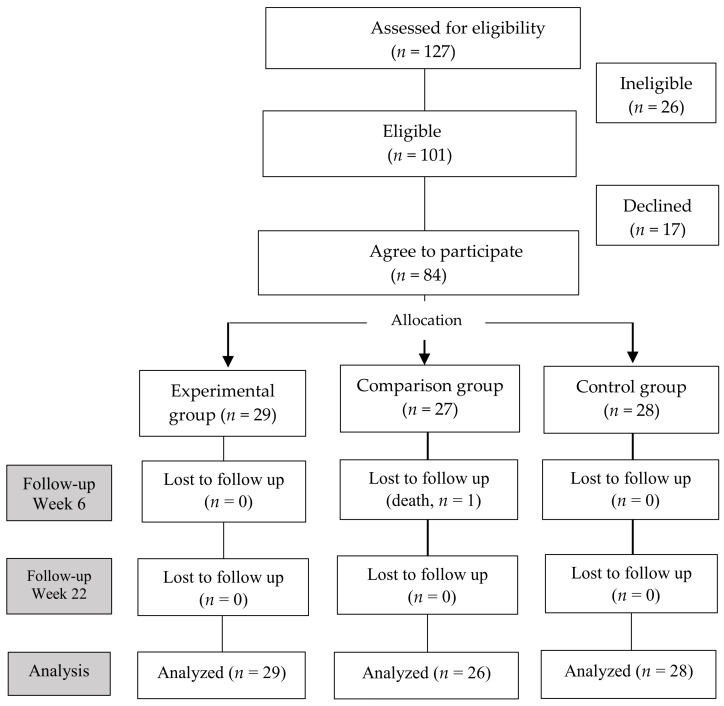
Flowchart of study.

The sample size was determined using G*Power program Ver.3.1.The calculation was set for repeated measures analysis of variance with an effect size of 0.16, a significance level of 0.05, a power of 0.80, three groups, and three repetitions. We set the effect size with reference to a previous study that examined the effectiveness of acupressure on saliva flow rate and xerostomia in HD patients [3]. The minimum required sample size was 81; however, we decided to recruit 89 participants, considering a 10% withdrawal rate. From January to July 2019, the managers of the HD units forwarded lists of patients who met the selection criteria. A total of 127 patients showed interest in the study. Of those, 101 met the inclusion criteria and were invited to participate. After 84 patients consented to participate, 29, 27, and 28 patients were selected for the experimental, comparison, and control groups, respectively (Figure 1).

### 2.4. Ethical Consideration

This study was approved by the institutional review board at K University Hospital (KHNMC-07-025-001). All participants joined the study after providing written consent and were given the freedom to withdraw from the study at any time.

### 2.5. Intervention Program

This 6-week intervention program included a fluid-restriction adherence program and AA. The experimental and comparison groups attended the 60-min fluid-adherence program once a week for 6 weeks. The experimental group received additional AA at three auricular acupoints for 6 weeks. 

#### 2.5.1. Fluid-Restriction Adherence Program

This was developed with reference to the recommendations of the National Kidney Foundation for HD patients to improve fluid-restriction adherence [7]. The fluid-restriction adherence program consisted of individual counseling, group education, and group discussion. The individual and group sessions were conducted in a meeting room and a small conference room next to the HD unit by two Ph.D.-level RNs for 60 min per week.

(1) Individual counseling: Each participant underwent face-to-face counseling with the RN for about 10 min before each group session. In an attempt to facilitate fluid-tracking and provide personalized fluid goals, participants submitted their diet and fluid intake logs and reviewed the records with the RN. They were encouraged to set their fluid intake goals for the following week. Participants were provided with a printout of the contracts and were encouraged to post the printout on an easily noticeable spot at home.
(2) Group education: A 20-min group lesson regarding the dietary sources of fluid, salt restriction strategies, ways to alleviate dry mouth without increasing fluid intake, self-monitoring of the amount of urine output and fluid intake, and problem-solving skills for situations leading to non-adherence was provided by the RN. Participants received fluid-restriction adherence booklets and a one-page summary of the information learned every week; they were encouraged to post these summaries next to their fluid intake contracts to remind themselves of ways to limit their fluid intake.
(3) Group discussion: Participants were divided into small groups of 5–8 people. The RNs served as facilitators to encourage patients to actively participate in the group discussion for 30 min. By discussing difficulties associated with fluid-restriction and experiences of successful adherence with their peers, the participants could improve their confidence and social support to continue their fluid-restriction.

#### 2.5.2. Auricular Acupressure

**Figure 2:**
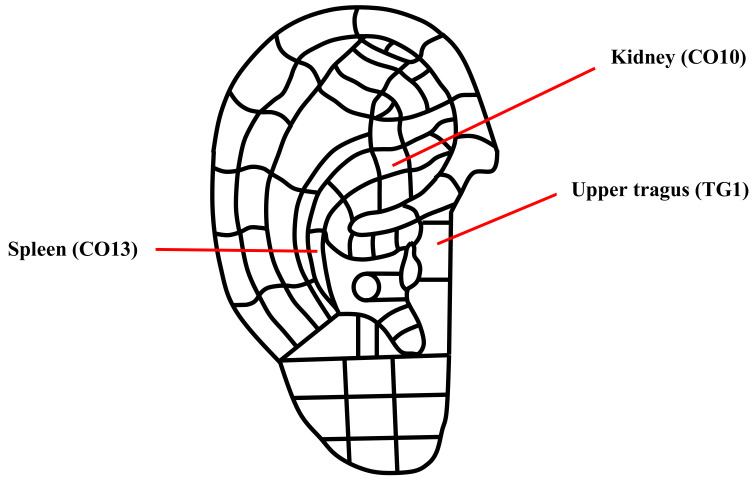
A map of the three auricular acupoints used in this study.

The protocol for AA was based on previous literature [14,20]. The AA was conducted by an RN, who is certified as an AA therapist, while participants underwent HD. The auricular acupoints chosen were as follows: kidney (CO10), spleen (CO13), and upper tragus (TG1; Figure 2). The kidney and spleen points are in the triangular fossa, where the auricular points for the vagus nerve and auricular branch for the auriculotemporal nerve are distributed; these nerves play a crucial role in activating the parasympathetic nerves, which can increase saliva production. In addition, the upper tragus is effective for saliva secretion and thirst [14]. Based on the 6-week pilot investigation of three HD patients, the finger pressure was set to 0.3–0.5 kg. After disinfecting the site for AA with 70% alcohol, one sticker containing one seed of semen vaccaria was attached to each of the three acupoints. The participants were taught to press on the acupoints three times a day and whenever they felt dry mouth at home. The RN replaced the sticker at every HD session.

### 2.6. Control Group

The control group received the usual renal care. They were also provided a fluid-restriction adherence booklet and a diary to record their fluid intake at home.

### 2.7. Measurements

#### 2.7.1. Salivary Flow Rate

The unstimulated whole saliva flow rate was measured. Saliva was collected using a cotton roll kept in the mouth for 5 min. Saliva samples were collected during HD at least 1 h after eating, smoking, or drinking. An electronic scale (CAS 120; Seoul, Korea) with 0.0001 g precision was used. The cotton weight was subtracted from the measurement, and the saliva weight was converted to the amount of secretion per minute (mL/min).

#### 2.7.2. Xerostomia

A visual analog scale from 0 (none) to 10 (severe) was used to measure xerostomia.

#### 2.7.3. Interdialytic Weight Gain

IDWG refers to the difference (kg) between the pre- and post-dialysis body weight. The pre-dialysis body weight was measured within 30 min of beginning dialysis, using an electronic scale (CAS, 200B; Seoul, Korea), and the post-dialysis body weight was measured within 30 min of the completion of dialysis, using the same scale. The weekly average IDWG value was considered.

#### 2.7.4. Fluid Control

The 24-item Fluid Control in Hemodialysis Patient Scale by Cosar and Pakyuz [21] was used. It assesses the knowledge, behavior, and attitude about fluid-restriction. Each item is rated on a 3-point Likert scale, and a higher score indicates better fluid-restriction adherence. Cronbach’s α was 0.88 at the time of scale development and was 0.82 in our study.

#### 2.7.5. Diet-Related Quality of Life

The 25-item Korean version of the Quality of Life Related to Dietary Change Questionnaire, an adapted version of the original tool developed by Delahanty, Hayden, Ammerman, and Nathan [22], was used to measure DQOL. The instrument assesses the QOL related to dietary change, satisfaction, and dietary impact. Each item is rated on a 4-point Likert scale, and a higher score indicates a higher DQOL. The Cronbach’s α was 0.79 in the study by Lee, Kim, and Kim [23] and was 0.85 in this study.

### 2.8. Statistical Analysis

Data were analyzed using IBM SPSS Statistics for Windows (Version 23.0. Armonk, NY, USA, IBM Corp). Homogeneity among the three groups was analyzed using chi-square tests, Fisher’s exact tests, and one-way analyses of variance. Considering the correlated structure of data from repeated measures at baseline and at 6- and 22-weeks, a generalized estimating equation (GEE) was used for analysis. Changes across time points for each group were analyzed using the Friedman test.

## 3. Results

### 3.1. Demographic and Baseline Characteristics

Of the 84 participants, one participant in the comparison group died during the study period and did not complete the post-program survey. Participants’ mean age was 64.2 ± 13.5 years, and 54.8% were men. The rest of demographic and baseline data are shown in Table 1. There were no significant differences in the general characteristics and outcome variables among the three groups at baseline (Table 1).

### 3.2. Salivary Flow Rate following the Intervention

While the experimental group showed significant increases in the salivary flow rate (*p* = 0.009), the comparison and control groups did not show significant changes in the salivary flow rate across the study period (Table 2). A GEE model showed that there was a significant time-and-group interaction for the salivary flow rate (*χ*^2^ = 26.60, *p* < 0.001; Table 3). Compared with the control group, the experimental group showed significant improvements in the salivary flow rate at weeks 6 and 22 (both *p* < 0.001); however, there were no significant differences in the comparison group at any of the follow-up assessments (Table 3).

### 3.3. Xerostomia following the Intervention

A GEE model did not show a significant time-and-group interaction for xerostomia (*χ*^2^ = 2.21, *p* = 0.696; Table 3).

### 3.4. Fluid Control following the Intervention

The experimental and comparison groups showed significant improvements in the fluid control score (*p* < 0.001 and *p* = 0.001, respectively); however, there were no significant changes in the fluid control score in the control group across the study period (Table 2). A GEE model revealed a significant interaction between time and group for fluid control (*χ*^2^ = 37.00, *p* < 0.001; Table 3). Compared with the control group, the experimental group showed a significant improvement in fluid control at weeks 6 and 22 (*p* = 0.048 and *p* < 0.001, respectively). However, the comparison group had a significantly higher fluid control than the control group at week 6 (*p* = 0.006; Table 3).

### 3.5. Interdialytic Weight Gain Following the Intervention

In the experimental group, IDWG decreased significantly across the study period (*p* = 0.003). However, the control group showed a significant increase in IDWG (*p* = 0.048; Table 2). There was a significant time-and-group interaction for IDWG (*χ*^2^ = 20.50, *p* < 0.001; Table 3). The experimental group had significantly lower IDWG than the control group after 6 weeks and 4 months (*p* = 0.003 and *p* < 0.001, respectively). No significant differences in IDWG between the comparison and control group were found at any of the follow-up assessments (Table 3). 

### 3.6. Diet-Related Quality of Life Following the Intervention

The experimental group showed a significant increase in DQOL across the three time points (*p* < 0.001). However, the control group showed a significant decline in the DQOL over time (*p* = 0.008: Table 2). There was a significant interaction between time and group for DQOL (*χ*^2^ = 16.26, *p* = 0.003; Table 3). Compared with the control group, the experimental group showed a significant improvement in DQOL at weeks 6 and 22 (both *p* < 0.001); however, the comparison group only had a higher DQOL score than the control group at week 6 (*p* = 0.028; Table 3). 

## 4. Discussion

This study demonstrated that a combined use of AA and a fluid-restriction adherence program significantly improved salivary flow rate, fluid control, IDWG, and DQOL of HD patients. Given that a leading cause of mortality and morbidity among this population is related to fluid overload, the improvement and maintenance of fluid management is clinically meaningful.

The average baseline salivary flow rate of our participants was 0.08 mL/min, which indicated hyposalivation. This result suggests that our participants may have experienced xerostomia and had difficulty chewing dry food, which led to drinking fluids to help them swallow. To avoid xerostomia symptoms, saliva needs to be secreted at a minimum rate of 0.1 mL/min [6]. While comparison and control groups demonstrated a decline in salivary flow rate throughout the study period, the experimental group showed a significant improvement in the salivary flow rate above 0.11 mL/min at 4 months post-intervention. This was in line with previous findings, which reported that acupressure on the ear and neck for 4 weeks significantly increased the salivary flow rate in HD patients [3]. The three acupoints used in this study were also used in another AA study on thirst and xerostomia in HD patients; these points are related to the regulation of the vagus nerve [14]. Stimulation of the auricular vagus nerve seems to result in an increased salivary flow rate via secretion of neurotransmitters such as neuropeptide Y, substance P, calcitonin gene-related peptides, and neurokinin A [6]. A previous study reported that at least 4 weeks of acupressure treatment is needed to stimulate saliva secretion in HD patients [3]; however, that study did not conduct a follow-up assessment and could not examine the long-term effects of AA. Thus, we present evidence showing that increased saliva secretion is maintained for 4 months after 6 weeks of AA. Subsequent studies should examine whether the effect of AA on saliva secretion is maintained over the long-term.

We found that xerostomia was not affected by the intervention program and revealed no significant changes between the groups. This is similar to a finding that thirst and xerostomia did not change significantly after a 5-week psychological intervention [10], and to the report that the AA did not significantly improve thirst symptom when compared to the sham-AA [3]. Since all participants in this study were provided with a fluid-restriction adherence booklet that included methods to alleviate xerostomia, this information may have contributed to the insignificant difference in the perception of xerostomia between the groups.

Compared to the comparison and control groups, the experimental group yielded benefits in the level of fluid control and IDWG. Our results align with the previous findings indicating that the combination of 6 weeks of muscle relaxation exercises and an empowerment program resulted in significant improvements in decision-making, stress reduction, positive attitudes, and IDWG in HD patients [9]. However, our results contradict those of other studies wherein a fluid distribution timetable did not significantly lower IDWG [11], and the IDWG, blood pressure, and biochemical parameters did not change significantly after the AA program [14]. A solitary intervention often fails to refrain HD patients from excessive water intake [24]. Our program provided a multifaceted intervention to improve fluid control. In particular, individual counseling and group discussion helped participants control dietary salt and fluid intake and reduced xerostomia through AA, which helped them maintain their fluid allowance. According to a recent systematic review, the combination of theory and patient health intervention may be more beneficial to improve fluid-restriction adherence in HD patients [4,17]. It is likely that empowerment strategies, such as the enhancement of self-control through tracking fluid intake, knowledge enhancement from patient-centered education, skill training through AA, and social support from other patients and RNs, may change the participants’ fluid intake behavior, which, in turn, leads to reduced IDWG.

Diet-related quality of life refers to a state of well-being influenced by diet that is assessed from social and psychological aspects [25], and it is significantly correlated with the health-related quality of life among patients undergoing HD [23]. Patients with ESRD experience substantial pressure regarding the recommended renal diet owing to restriction of food and fluid, the financial burden, and difficulties with meal preparation [17,18,25]. The DQOL was significantly lower in this population than in patients with other chronic diseases [23]. Our program significantly improved the DQOL of the experimental group, which is similar to the previous report that a diet management program including individual counseling, group education, and group discussion had a positive impact on the DQOL [25] and that an empowerment program leads to increased QOL for HD patients [9]. Considering that reduced saliva and xerostomia are negatively correlated with quality of life among patients with ESRD [6,11], alleviating xerostomia by increasing the salivary flow rate in EG may have increased the participants’ DQOL. A previous systematic review revealed that existing studies on fluid-adherence interventions did not include psychosocial outcome measures such as QOL and well-being, and it is impossible to draw conclusions about the effect of the interventions on quality of life [16]. However, the improvement in DQOL following our program may serve as evidence that improving fluid-adherence is an effective means of increasing the QOL of HD patients.

There were some limitations to our study. First, our conclusion is not definitive because of the small sample size. Further studies with larger samples are recommended. Second, the individuals administering the intervention were not blinded to the treatment conditions; thus, the potential for bias exists. Third, we enrolled only Koreans who had been admitted to three HD centers in one of the largest cities in Korea. Thus, the results may not be generalized to different populations.

## 5. Conclusions

A combined use of AA and a fluid-restriction adherence program had beneficial effects on improving salivary flow rate, fluid control, IDWG, and DQOL. The significant improvements were retained at the 22-week follow-up, suggesting that adding AA to a fluid-adherence program has a stronger and longer impact on improving fluid control and quality of life compared to only providing a fluid-restriction intervention to HD patients. Therefore, health-care professionals working in HD units may adopt this program as a cost-effective, safe, and complementary tool to promote sustainable patient adherence to fluid-restriction.

## Figures and Tables

**Table 1:** Comparison of participants’ demographics and outcome variables (*n* = 84).

Variable	Experimental Group	Comparison Group	Control Group	χ^2^/*F*	*p-*Value
	(*n* = 29)	(*n* = 27)	*(n* = 28)		
	*n* (%)/M ± SD	*n* (%)/M ± SD	*n* (%)/M ± SD		
Gender (male)	12 (44.4)	18 (64.3)	16 (55.2)	2.18	0.33
Age (years)	67.4 ± 11.4	61.8 ± 14.1	63.2 ± 14.9	1.30	0.27
Education					
Primary school	6 (22.2)	8 (28.6)	6 (20.7)	2.41 ^1^	0.87
Middle school	2 (7.4)	2 (7.1)	2 (6.9)		
High school	13 (48.1)	10 (35.7)	16 (55.2)		
≥College	6 (22.2)	8 (28.6)	5 (17.2)		
Marital status (married)	16 (59.3)	18 (64.3)	19 (65.5)	7.86	0.24
Number of comorbidities	1.6 ± 1.4	2.0 ± 1.3	1.8 ± 1.1	0.53	0.59
Diabetes mellitus (yes)	12 (42.9)	14 (51.9)	10 (34.5)	1.72	0.42
Hemodialysis vintage (months)	55.7 ± 41.3	60.9 ± 53.4	46.4 ± 47.7	0.65	0.52
Smoking (no)	25 (92.6)	19 (67.9)	24 (82.8)	5.54	0.06
Alcohol (no)	24 (88.9)	23 (82.1)	23 (79.3)	0.96	0.61
Vascular access type					
AVF	26(89.7)	27(100.0)	23(82.1)	5.121	0.08
AVG	3(10.3)	0(0.0)	5(17.9)		
Parathyroid hormone (pg/mL)	280.8 ± 156.5	354.1 ± 122.4	324.7 ± 166.2	2.200	0.117
Urine output (mL/day)	169.2 ± 206.2	159.21 ± 177.3	186.8 ± 218.3	1.077	0.345
Salivary flow rate (mL/min)	0.08 ± 0.04	0.09 ± 0.05	0.08 ± 0.05	0.42	0.65
Xerostomia	4.83 ± 2.71	5.33 ± 2.34	4.71 ± 2.72	0.80	0.45
Fluid control	50.32 ± 6.04	51.22 ± 5.81	52.61 ± 6.24	0.19	0.82
Interdialytic weight gain (kg)	2.07 ± 0.86	1.87 ± 0.87	1.98 ± 0.84	0.06	0.93
Diet-related quality of life	61.86 ± 11.05	62.93 ± 9.06	65.46 ± 9.62	0.18	0.83

^1^ Fisher’s exact test; M: mean, SD: standard deviation, AVF: arteriovenous fistula, AVG: arteriovenous graft.

**Table 2:** Effects of the intervention program on study outcomes.

Variable	Baseline	Week 6	Week 22	χ^2^	*p-*Value ^1^
	M ± SD	M ± SD	M ± SD		
Salivary flow rate (mL/min)					
Experimental group	0.08 ± 0.04	0.10 ± 0.06	0.11 ± 0.05	9.376	0.009
Comparison group	0.09 ± 0.05	0.09 ± 0.05	0.08 ± 0.05	2.509	0.28
Control group	0.08 ± 0.05	0.08 ± 0.04	0.07 ± 0.03	4.450	0.10
Xerostomia					
Experimental group	4.83 ± 2.71	4.24 ± 2.87	4.29 ± 2.46	0.306	0.85
Comparison group	5.33 ± 2.34	5.26 ± 1.81	5.22 ± 2.00	0.812	0.66
Control group	4.71 ± 2.72	4.93 ± 2.49	4.89 ± 2.56	0.674	0.71
Fluid-restriction adherence					
Experimental group	50.32 ± 6.04	53.71 ± 6.2	58.11 ± 8.27	23.792	<0.001
Comparison group	51.22 ± 5.81	55.00 ± 5.13	52.00 ± 5.33	13.118	0.001
Control group	52.61 ± 6.24	53.07 ± 4.57	50.32 ± 5.08	4.891	0.08
Interdialytic weight gain (kg)					
Experimental group	2.06 ± 0.87	1.60 ± 0.59	1.50 ± 0.87	11.345	0.003
Comparison group	1.87 ± 0.87	1.64 ± 0.75	1.73 ± 0.74	4.178	0.12
Control group	1.98 ± 0.84	1.94 ± 0.82	1.99 ± 0.85	6.065	0.048
Diet-related Quality of Life					
Experimental group	61.86 ± 11.05	65.52 ± 9.67	66.32 ± 9.16	9.048	0.011
Comparison group	62.93 ± 9.06	65.3 ± 9.5	60.19 ± 7.50	2.272	0.32
Control group	65.46 ± 9.62	62.89 ± 8.02	58.39 ± 7.69	9.761	0.008
Parathyroid hormone (pg/mL)					
Experimental group	276.48 ± 160.81	288.79 ± 135.27	258.09 ± 157.39	4.519	0.104
Comparison group	349.85 ± 122.77	377.08 ± 136.89	380.37 ± 142.34	3.769	0.152
Control group	324.67 ± 166.18	341.84 ± 167.23	348.07 ± 206.47	2.643	0.267
Urine output (mL/day)					
Experimental group	169.23 ± 206.21	160.60 ± 239.49	168.09 ± 246.40	1.380	0.502
Comparison group	159.21 ± 177.31	151.14 ± 211.61	151.45 ± 200.63	1.595	0.450
Control group	186.82 ± 218.28	182.08 ± 216.58	164.21 ± 189.46	5.072	0.079

^1^ Friedman test; M: mean, SD: standard deviation.

**Table 3:** Generalized estimating equation analysis of salivary flow rate, xerostomia, fluid-restriction adherence, interdialytic weight gain and diet-related quality of life.

	Estimate	Standard Error	Wald χ^2^	*p-*Value
Salivary flow rate ^1^				
Experimental group	−0.07	0.15	0.246	0.62
Comparison group	0.07	0.14	0.216	0.64
Time (week 6)	−0.03	0.03	1.377	0.24
Time (week 22)	−0.11	0.05	5.513	0.019
Experimental group × time (week 6)	0.25	0.07	13.769	<0.001
Experimental group × time (week 22)	0.45	0.09	22.891	<0.001
Comparison group × time (week 6)	0.04	0.05	0.552	0.45
Comparison group × time (week 22)	0.07	0.07	1.003	0.31
Xerostomia^2^				
Experimental group	−0.02	0.12	0.019	0.89
Comparison group	0.08	0.11	0.450	0.50
Time (week 6)	0.01	0.10	0.004	0.94
Time (week 22)	0.01	0.07	0.019	0.89
Experimental group × time (week 6)	−0.12	0.15	0.630	0.42
Experimental group × time (week 22)	−0.20	0.13	2.123	0.14
Comparison group × time (week 6)	−0.07	0.11	0.379	0.53
Comparison group × time (week 22)	−0.08	0.12	0.470	0.49
Fluid control ^3^				
Experimental group	−0.04	0.03	1.964	0.16
Comparison group	−0.03	0.03	0.754	0.38
Time (week 6)	0.01	0.02	0.338	0.51
Time (week 22)	−0.04	0.03	3.211	0.07
Experimental group × time (week 6)	0.06	0.03	3.921	0.048
Experimental group × time (week 22)	0.20	0.04	30.844	<0.001
Comparison group × time (week 6)	0.62	0.02	7.691	0.006
Comparison group × time (week 22)	0.06	0.03	3.498	0.06
Interdialytic weight gain ^4^				
Experimental group	0.09	0.22	0.167	0.68
Comparison group	−0.10	0.23	0.213	0.64
Time (week 6)	−0.04	0.04	0.873	0.35
Time (week 22)	0.02	0.05	0.084	0.77
Experimental group × time (week 6)	−0.44	0.15	9.118	0.003
Experimental group × time (week 22)	−0.58	0.14	17.970	<0.001
Comparison group × time (week 6)	−0.19	0.12	2.480	0.11
Comparison group × time (week 22)	−0.16	0.12	1.726	0.18
Diet-related Quality of Life ^5^				
Experimental group	−0.06	0.04	1.774	0.18
Comparison group	−0.04	0.04	1.055	0.30
Time (week 6)	−0.04	0.02	3.123	0.07
Time (week 22)	−0.11	0.03	12.684	<0.001
Experimental group × time (week 6)	0.10	0.04	7.170	0.007
Experimental group × time (week 22)	0.18	0.05	13.017	<0.001
Comparison group × time (week 6)	0.08	0.04	4.835	0.028
Comparison group × time (week 22)	0.07	0.04	2.603	0.10

^1^ group × time interaction *χ*^2^ = 26.60, *p* < 0.001. ^2^ group × time interaction *χ*^2^ = 2.21, *p* = 0.69. ^3^ group × time interaction *χ*^2^ = 37.00, *p* < 0.001. ^4^ group × time interaction *χ*^2^ = 20.50, *p* < 0.001. ^5^ group × time interaction *χ*^2^ = 16.26, *p* = 0.003.

## Data Availability

No new data were created or analyzed in this study. Data sharing is not applicable to this article.
